# Cyclic two-step electrolysis for stable electrochemical conversion of carbon dioxide to formate

**DOI:** 10.1038/s41467-019-11903-5

**Published:** 2019-09-02

**Authors:** Chan Woo Lee, Nam Heon Cho, Ki Tae Nam, Yun Jeong Hwang, Byoung Koun Min

**Affiliations:** 1 Department of Materials Science and Engineering, Seoul National University, Seoul, 08826 Republic of Korea; 2 Clean Energy Research Center, Korea Institute of Science and Technology, Seoul, 02792 Republic of Korea; 3 Department of Chemistry, Kookmin University, Seoul, 02707 Republic of Korea; 4 Department of Chemical and Biomolecular Engineering, Yonsei University, Seoul, 03722 Republic of Korea; 5 Green School, Korea University, Seoul, 02841 Republic of Korea

**Keywords:** Electrocatalysis, Hydrogen fuel

## Abstract

Pd metal and Pd-based alloys are ideal catalysts that allow for the electrochemical conversion of CO_2_ to HCOO^−^ at almost zero-overpotential with high selectivity, but catalyst degradation caused by concurrent CO poisoning limits their practical implementation. Here, we demonstrate that cyclic two-step electrolysis, by applying the reduction and oxidation potentials alternately, achieves 100% current density stability and 97.8% selectivity toward HCOO^−^ production for at least 45 h. The key idea for achieving the reliability is based on the selective removal of CO by controlling the parameters during the oxidation step, which utilizes the different reversibility of HCOO^−^ and CO production reactions. Furthermore, it is found that potentiostatic electrolysis causes CO adsorption and subsequent dehydridation, which in turn lowers HCOO^−^ selectivity. Our work provides a system-level strategy for solving the poisoning issue that is inevitable in many electrocatalytic reactions.

## Abstract (web summary)

Reduction of carbon dioxide is promising for the production of value-added chemicals, but electrocatalysts are hindered by carbon monoxide poisoning. Here, the authors alternate reduction and oxidation potentials to achieve stable and selective long-term electrocatalytic reduction of carbon dioxide to formate.

## Introduction

Electrochemical CO_2_ reduction combined with renewable energy resources represents one of the promising strategies for not only reducing greenhouse gas emissions, but also storing electrical energy in a value-added chemical form^1–4^. Among the products, HCOO^−^ is considered as a valuable liquid chemical for hydrogen storage because it can release H_2_ fuel responsibly at room temperature^5,6^. Pd metal is the only electrocatalyst that produces HCOO^−^ at zero-overpotential with high selectivity^7–13^. It has the special character because CO_2_ can be activated by palladium hydride (PdH_x_), which is cathodically formed in an aqueous electrolyte^14,15^. However, a key challenge is how to minimize or prevent catalyst degradation caused by CO poisoning^14^. Previous approaches have focused mostly on developing catalysts that favor HCOO^−^ production over CO adsorption^16–18^. These approaches were mostly based on the theoretical result that HCOO^−^ and CO are primarily formed from different intermediates, formate (OCHO*) and carboxylate (COOH*), respectively^19,20^. Although the degradation rate was lowered via catalyst development, the stability issue has not been fully resolved yet.

Two-step electrolysis, applying different potentials alternately, has been exploited as an easy and scalable method for decoupling concurrent multiple reactions in electroplating and electrochemical devices^21–23^. For example, in electrodepositing metals into porous templates^24,25^, though metal deposition and ion consumption occurred simultaneously, the ion concentration can be recovered by adding another potential step. As a result, the ion depletion effect can be excluded in one cycle unit. In addition, it has already been demonstrated that a reverse bias in the solid-oxide electrochemical cell (SOC) can restore the oxygen electrode degradation^22^. By cycling forward and reverse bias, stable performances can be achieved. Likewise, when a perovskite solar cell operates via day/night cycling, halide defects, which are the origin of performance loss, are created during irradiation and are eliminated at night ^23^.

Here, we discuss the degradation mechanism using electrochemical surface analysis, and based on the understanding, we propose cyclic two-step electrolysis, applying reduction and oxidation potentials alternately, as a strategy for securing both stability and selectivity for HCOO^−^ production. In CO_2_ reduction reactions on Pd-based metals, the adsorption and desorption of the CO intermediate can be reversible on the surface, whereas HCOO^−^ formation is irreversible because HCOO^−^ is released into the bulk electrolyte. Given that two-step electrolysis only affects reversible behaviors, the additional oxidation step can be used specifically to control the CO intermediate oxidation on the electrode. In addition to the reversibility issue, the different oxidation potentials of HCOO^−^ and CO provide another knob for tuning the selective reaction during the reverse cycle. CO oxidation mainly occurs between 0.82 and 1.22 V vs. RHE, whereas HCOO^−^ oxidation is blocked above 0.80 V vs. RHE. In setting the reverse potential, such a significant difference suggests that HCOO^−^ oxidation can be prevented even at high concentration if the reverse potential exceeds 0.80 V vs. RHE. Based on these principles, two-step electrolysis can decouple the HCOO^−^ formation from CO adsorption by selectively oxidizing CO. As a result, 100% of the initial current density is maintained even after 45 h, and a high HCOO^−^ selectivity of 97.8% is achieved.

## Results

### Catalyst synthesis and electrochemical surface analysis

As a model catalyst for stable and selective HCOO^−^ production, Pd_80_Ag_20_/C catalyst was synthesized and investigated, based on our observation that Pd_80_Ag_20_/C is more suitable in intrinsic properties than pristine Pd/C for improved reliability of HCOO^−^ production (Supplementary Fig. 1).

For the synthesis, microwave-assisted polyol synthesis and subsequent cyclic voltammetry (CV) pretreatment were performed. In the polyol method, commercial Pd/C powder was heat-treated via microwave irradiation at approximately 200 °C with the Ag precursor. X-ray diffraction (XRD) patterns of the as-synthesized powders (Supplementary Fig. 2) showed a clear peak shift compared to that of Pd/C due to alloying with Ag. The input molar ratio of Ag/Pd was adopted as 0.67, because the largest peak shift was observed without the formation of Ag crystals (Supplementary Fig. 2). After the polyol synthesis, CV pretreatment was applied to the as-synthesized nanoparticles (NPs). Anodic CV was scanned in Ar-purged 0.1 M HClO_4_ solution for four cycles (Supplementary Fig. 3). For as-synthesized Pd_80_Ag_20_/C, the irreversible Ag oxidation peak positioned at approximately 1.2 V vs. RHE was diminished with increasing cycle number, whereas the redox peak associated with reversible hydrogen cycling became distinct between 0.0 and 0.4 V vs. RHE. Additionally, the initial current density of electrolysis increased with the cycle number (Supplementary Fig. 4). From transmission electron microscopy (TEM) analysis (Supplementary Fig. 5), it was confirmed that Pd_80_Ag_20_/C catalysts are carbon-supported spherical NPs with an average size of ~5 nm. The composition was analyzed with inductively coupled plasma-optical emission spectrometry (ICP-OES) and scanning transmission electron microscopy-energy dispersive X-ray spectroscopy (STEM-EDS) (Supplementary Table 1, Figs. 6 and 7).

**Fig. 1:**
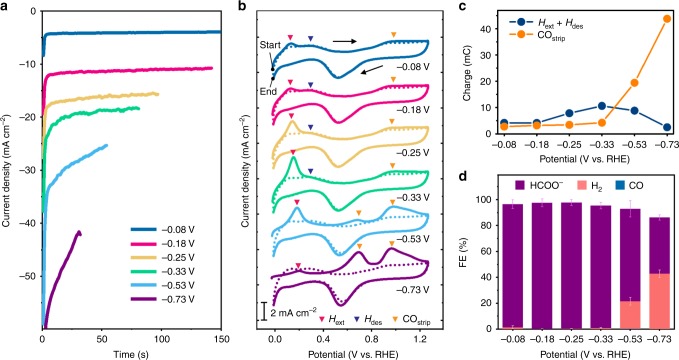
Relation between adsorbed/absorbed species and product selectivity. **a** Current density vs. time curves of Pd_80_Ag_20_/C catalysts at various potentials in a CO_2_-saturated electrolyte. In the electrolysis, passed charge is fixed at 0.8 C. **b** Cyclic voltammetry (CV) curves of Pd_80_Ag_20_/C catalysts recorded after electrolysis at various potentials. Solid and dotted lines are the first and second cycle of CV curves. In the first cycle, the anodic peaks related with H extraction (H_ext_) from the lattice, H desorption (H_des_) from the surface, and CO stripping (CO_strip_) are indicated with pink, blue, and orange triangles, respectively. **c** CO_strip_ and H_ext_ + H_des_ charges measured from the CV curves. **d** The Faradaic efficiencies (FEs) for H_2_, CO, and HCOO^−^ production measured from gas chromatograph (GC) and ^1^H nuclear magnetic resonance (NMR) analysis after electrolysis at various potentials in a gas-tight cell. Error bars: mean±s.d. Source data are provided as a Source Data file

Figure 1a shows the chronoamperometry curves of the Pd_80_Ag_20_/C catalysts measured at various potentials in a CO_2_-saturated 0.5 M NaHCO_3_/0.5 M NaClO_4_ electrolyte. The passed charge was fixed at 0.8 C. The Pd_80_Ag_20_/C catalyst showed a higher degradation rate of electrocatalytic current density at lower potentials. The degradation rate was measured to be 0.04, 0.08, 0.13, 0.26, 0.46 and 0.79% s^−1^ at −0.08, −0.18, −0.25, −0.33, −0.53, and −0.73 V vs. RHE, respectively. Basically, CO adsorption can be accelerated at more negative potentials because it is an electrochemical process, while the desorption of adsorbed CO is a chemical process, independent on potentials. Accordingly, the more negative potentials can cause the higher rate of CO poisoning, resulting in the faster degradation rate.

To deeply understand the observed trends, adsorbed chemical species and absorbed hydrogen were investigated via anodic cyclic voltammetry (CV) after electrolysis (Fig. 1b). The first and second cycles are denoted as solid and dotted lines, respectively. The anodic scan of the first cycle showed stripping peaks of H and CO as highlighted by triangle symbols (Fig. 1b). The anodic peaks at around 0.15 and 0.31 V vs. RHE can be assigned to H extraction (H_ext_) from the lattice and H desorption (H_des_) from the surface, respectively^26^. The oxidation peaks positioned at 0.97 and 0.69 V vs. RHE correspond to the oxidation of adsorbed CO at Pd(111) terrace and stepped sites^27–29^. The CO oxidation was also proved by comparing the anodic CV spectra recorded after applying a cathodic potential in Ar- and CO-purged electrolytes (Supplementary Fig. 8). Additionally, with the CO oxidation, metal oxidation also occurred from 0.70 to 1.22 V vs. RHE. In the cathodic scan of the first cycle, the oxidized metal was reduced again at 0.55 V vs. RHE, and then H adsorption and absorption occurred at 0.15 and 0.00 V vs. RHE, respectively. In the second cycle, the redox reactions related with the dehydridation/hydridation and the formation of oxide and metal were observed, whereas the CO oxidation peaks disappeared.

One of the most interesting features is that CO stripping (CO_strip_) peaks evolves as the potential decrease, and the H_ext_ and H_des_ peaks compete with the CO_strip_ peaks. In the CV spectra of Pd_80_Ag_20_/C (Fig. 1b), we observed that the H_ext_ peak gradually increases from −0.08 to −0.33 V vs. RHE but decreases at more negative potentials. The H_des_ peak also declined distinctly with the decrease of H_ext_ peak. The decrease of the H_ext_ and H_des_ peaks was accompanied by the steep increase of CO_strip_ peaks. A similar trend was also observed in the pristine Pd/C case (Supplementary Fig. 9). As the potential became more negative from −0.08 to −0.33 V vs. RHE, the H_ext_ and H_des_ peaks decreased and ultimately disappeared whereas the CO_strip_ peaks increased dramatically. Because the anodic peaks indicate the oxidation of chemical species adsorbed and absorbed by electrolysis, these results show that lower potentials lead to the increase of CO adsorption and the decrease of H adsorption/absorption amount.

### Relation between adsorbed/absorbed species and product selectivity

To determine how the surface chemical species affects CO_2_ reduction, we compared the amount of surface species and product selectivity as a function of potential. Figure 1c shows the CO_strip_ and H_ext_ + H_des_ charges calculated by integrating the area under the oxidation peaks of the anodic CV spectra. The CO_strip_ and H_ext_ + H_des_ charges reflect the amount of adsorbed CO and absorbed/adsorbed H during electrolysis, respectively. The product selectivity was investigated by a gas chromatograph (GC) and a ^1^H nuclear magnetic resonance (NMR) spectrometer by sampling the headspace gaseous mixtures and the electrolyte after electrolysis (Fig. 1d and Supplementary Fig. 10). The Faradaic efficiency (FE) for HCOO^−^ accounted for 96.1% on average from −0.08 to −0.33 V vs. RHE and declined abruptly at lower potentials with the increase of H_2_ evolution. In contrast, CO was detected in a negligible amount with an average FE of 0.1%. Considering that the amount of CO adsorption also dramatically increased below −0.33 V vs. RHE (Fig. 1c), it can be empirically shown that there is a correlation between CO adsorption and the increase in H_2_ evolution. Specifically, the CO_strip_ charges were 3.4, 4.2, 19.4 and 43.8 mC at −0.25, −0.33, −0.53 and −0.73 V vs. RHE, respectively, while the FEs for H_2_ production were measured to be 0.2%, 0.7%, 21.3% and 42.7% at the examined potentials. A similar trend can be also observed in the Pd/C case (Supplementary Fig. 11). The decrease in the total FE for H_2_, CO and HCOO^−^ products at high overpotentials can be attributed to CO adsorption, H adsorption/absorption and non-Faradaic process (Supplementary Table 2).

Taken together, CO adsorption contributes to decreasing the amount of absorbed/adsorbed H and increasing H_2_ evolution, while deeper insights into CO_2_ reduction reactions on Pd-based catalysts can be discussed based on theoretical consideration of intermediate binding. According to a previous calculation study^30^, it is known that CO adsorption lowers the binding strength of the H intermediate. The weaker binding of H can increase H_2_ evolution and accelerate dehydridation, causing the decrease in HCOO^−^ selectivity, which is demonstrated in this study. This phenomenon implies that intrinsically strong H binding of Pd-based metals enables high selectivity for HCOO^−^ formation. However, it should not be overlooked that the H binding energy is also correlated with CO binding energy^19^. Strong H binding leads to strong CO binding. Consequently, it is challenging to completely separate the HCOO^−^ production and CO poisoning in potentiostatic electrolysis owing to the complex interrelations.

### Cyclic two-step electrolysis

**Fig. 2:**
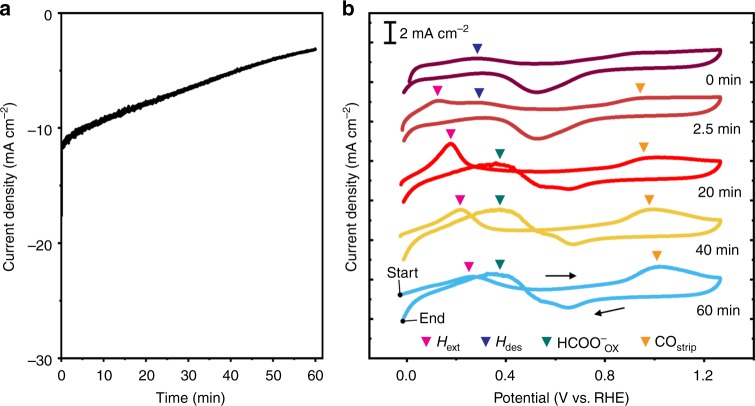
Electrochemical oxidation behavior of HCOO^−^ and CO products. **a** Current density vs. time curve of Pd_80_Ag_20_/C catalysts recorded during 1 h electrolysis. Chronoamperometry was conducted at −0.18 V vs. RHE in a CO_2_-saturated electrolyte. The current density gradually decreases with time. **b** Cyclic voltammetry curves measured after electrolysis for different durations at −0.18 V vs. RHE. The anodic peaks associated with H extraction (H_ext_), H desorption (H_des_), HCOO^−^ oxidation (HCOO^−^_ox_), and CO stripping (CO_strip_) are indicated by pink, blue, green, and orange triangles, respectively

We applied cyclic two-step electrolysis to completely resolve the stability issue using an extrinsic system-level control. The cyclic two-step electrolysis involves the programmed alternation of reduction and oxidation. Its special advantage is that HCOO^−^ and CO formation can be decoupled. The decoupling principle can be understood based on empirical findings from electrochemical surface analysis. Firstly, HCOO^−^ is released well into the bulk electrolyte while CO remains adhered to the catalyst surface (Fig. 1b, d). Due to the difference in adsorption behavior, adding an oxidation step after CO_2_ reduction can selectively induce the reverse reaction for CO adsorption. After the oxidation step, HCOO^−^ becomes the sole reaction product caused by the two-step electrolysis. This result means that HCOO^−^ production is decoupled from CO adsorption. Secondly, the HCOO^−^ and CO oxidations occurred in different potential regimes. When the stripping analysis was performed after electrolysis for various durations at −0.18 V vs. RHE (Fig. 2a, b), the CO oxidation peaks were observed between 0.82 to 1.22 V vs. RHE, whereas the HCOO^−^ oxidation was prevented at above 0.80 V vs. RHE. Thus, controlling the oxidation potential enables selective CO oxidation. Thirdly, the oxidation of adsorbed CO occurred preferentially before the initiation of HCOO^−^ oxidation (Fig. 2b). This result means that controlling the oxidation period enables selective CO oxidation. Based on these principles, we designed and optimized the potential program of cyclic two-step electrolysis.

**Fig. 3:**
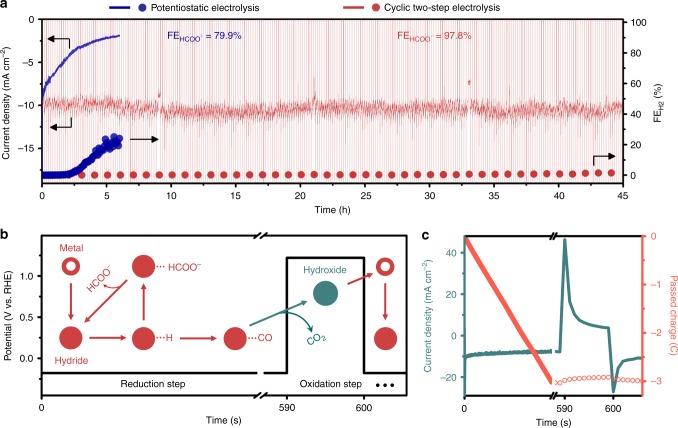
HCOO^−^ production performance of cyclic two-step electrolysis. **a** Comparison between cyclic two-step electrolysis and potentiostatic electrolysis in terms of the current density and product selectivity. The blue and red lines represent the current density vs. time curves measured via potentiostatic electrolysis and cyclic two-step electrolysis, respectively. The blue and red circles represent H_2_ production selectivity. Both experiments were conducted in a flow cell system where CO_2_-saturated bicarbonate solution was continuously refreshed using a peristaltic pump. **b** Potential program and reaction scheme of the cyclic two-step electrolysis. In the reduction step, −0.18 V vs. RHE was applied for 590 s, whereas 1.22 V vs. RHE was applied for 10 s in the oxidation step. **c** Current density vs. time curves and net passed charges during cyclic two-step electrolysis. The charge used in the oxidation step accounts for 3.7% of the charge passed in the reduction step

Figure 3a shows the time-dependent profiles of current density (solid line) and H_2_ production FE (solid circles) recorded during cyclic two-step electrolysis and potentiostatic electrolysis on Pd_80_Ag_20_/C catalysts. The FE for gas products was measured by GC analysis under a constant gas flow, whereas the average HCOO^−^ FE was obtained via ^1^H NMR analysis of the catholyte after electrolysis. In cyclic two-step electrolysis, the potential program was composed of a reduction and oxidation step per cycle as shown in Fig. 3b. In the reduction and oxidation step, −0.18 and 1.22 V vs. RHE were applied for 590 and 10 s, respectively. The redox cycle was repeated 270 times for 45 h. During the long-term experiments, the CO_2_-saturated electrolyte was continuously circulated using a peristaltic pump between electrolyte bottles and an electrochemical cell (Supplementary Fig. 12).

Surprisingly, the catalytic performance remained stable for 45 h via cyclic two-step electrolysis as shown in Fig. 3a. The initial current density of the 1st cycle (11.0 mA cm^−2^) was identical to that of the 270th cycle (11.0 mA cm^−2^), indicating that the current density loss is 0.0% during the long-term experiment. In addition, H_2_ production was suppressed to under 1.5% throughout the experiment. Consequently, an average HCOO^−^ FE of 97.8% was achieved. In detail, as displayed in the expanded current density profile (Fig. 3c), the initial current density of 11.0 mA cm^−2^ decreased to 7.8 mA cm^−2^ during the reduction step due to the CO accumulation. In the following oxidation step for 10 s, the cathodic current was switched to an anodic current, and the value rapidly decayed to nearly zero in a few seconds. During the anodic step, the oxidation of adsorbed CO and hydride predominantly occurred. Afterward, in the next reduction step, the current polarity was inverted again and the current density was dramatically restored to 11.4 mA cm^−2^. The degradation and recovery of current density were repeated in every cycle. As catalyst surface was regenerated continuously via the programmed anodic step, zero degradation of current density could be achieved regardless of electrolysis duration. In addition, as severe CO adsorption was prevented, very low selectivity for H_2_ production could be stably maintained. To the best of our knowledge, these catalytic performances show record-high stability in terms of current density and selectivity in comparison with the previous reports on HCOO^−^ production using Pd-based catalysts (Supplementary Table 3)^7,14,16–18,31–34^. In contrast, potentiostatic electrolysis showed a current density loss as large as 63.2% in only 6 h. Simultaneously, H_2_ evolution selectivity abruptly started to increase after 2 h, and reached to 24.1% after 6 h. As a result, the average HCOO^−^ FE for 6 h was measured to be 79.9%. The clear stability difference between the two experiments indicates that CO adsorption, the origin of catalyst degradation, was completely decoupled from HCOO^−^ production in the two-step electrolysis.

The decoupling mechanism of CO adsorption can be figured out from the reaction scheme occurring in each potential step as shown in Fig. 3b. In the reduction step, the formation of HCOO^−^ and CO proceeds concurrently on the hydride surface formed by H absorption, as identified by product and stripping analyses (Figs. 1d and 2b). Among the reduced products, CO molecules are accumulated at the catalyst surface, whereas HCOO^−^ is released immediately and diffused into the bulk electrolyte. In the subsequent oxidation step, adsorbed CO is preferentially oxidized (Fig. 2b), whereas HCOO^−^ oxidation is prevented. The net reaction for one cycle is only HCOO^−^ production.

Additionally, the economic feasibility of two-step electrolysis method was evaluated by investigating the additional charge and electrical energy used in the oxidation step. In calculating the electrical energy consumption, the overpotential applied at the counter electrode was assumed to be zero for convenience. For example, the voltages of the reduction and oxidation step were set to be 0.18 + 1.23 V and 1.22 + 0.00 V, respectively. The charge consumed in the oxidation step accounted for only 3.7% of the charge passed in the reduction step as shown in Fig. 3c. In addition, the electrical energy consumed in the oxidation step was 1.9% of that in the reduction step. This proves that there is almost no difference in energy consumption between potentiostatic and cyclic two-step electrolysis methods.

### Key parameters

To achieve high stability and selectivity for HCOO^−^ production via cyclic two-step electrolysis, the adsorbed CO should be oxidized fully and selectively in the oxidation step. Current density recovery can reflect whether the adsorbed CO is fully oxidized to CO_2_, whereas HCOO^−^ selectivity can reveal if the oxidation step is used only for CO oxidation. By measuring the current density vs. time curves and the average HCOO^−^ FE during electrolysis with different oxidation potentials and periods, optimal parameters of the oxidation step were drawn. In controlling the oxidation parameters, the reduction potential and period were fixed at −0.18 V vs. RHE and 20 min, respectively.

**Fig. 4:**
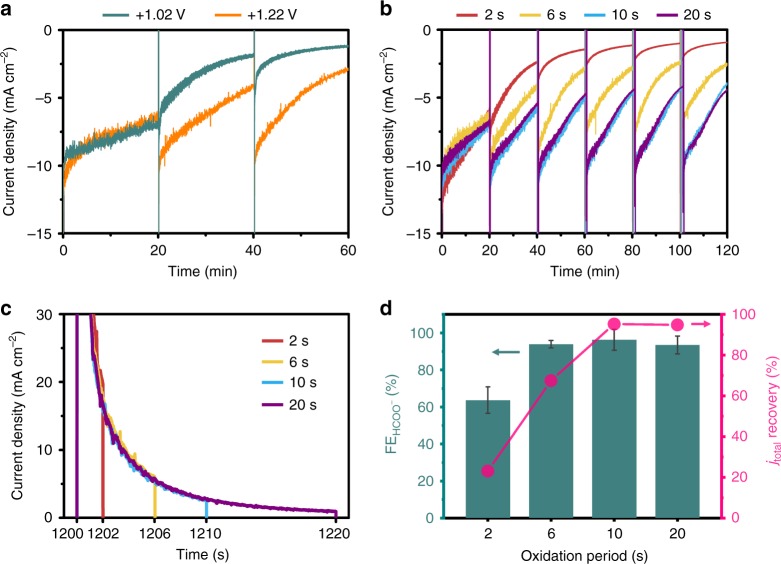
Key parameters of cyclic two-step electrolysis. **a** The effect of oxidation potential on current density recovery. The oxidation potential was changed with the oxidation period fixed at 6 s. **b** Current density vs. time curves as a function of oxidation period. The oxidation period was varied from 2 to 20 s while the oxidation potential was fixed at 1.22 V vs. RHE. In controlling the oxidation parameters, the reduction potential and period were fixed to −0.18 V vs. RHE and 20 min. **c** Current density vs. time curves of the oxidation step with various oxidation periods. **d** The dependence of the current density (*j*_total_) recovery and the HCOO^−^ Faradaic efficiency (FE) on the oxidation period. The HCOO^−^ FE was measured using ^1^H nuclear magnetic resonance (NMR) analysis of the liquid product after cyclic two-step electrolysis. The *j*_total_ recovery values were calculated by dividing the initial current density of the sixth cycle by that of the first cycle in Fig. 4b. Error bars: mean±s.d. Source data are provided as a Source Data file

Figure 4a shows the effect of the oxidation potential on current density recovery, where the oxidation period was fixed at 6 s. Oxidation potentials of 1.02 and 1.22 V vs. RHE were examined because CO oxidation can occur at these potentials as displayed in Fig. 2b. For the first cycle, the current densities similarly decreased with time at both oxidation potentials, whereas the current density difference became larger in the following steps. The current density recovery was calculated by dividing the initial current density of the third cycle by that of the first cycle. The current density recovery was 44.4% and 78.8% at 1.02 and 1.22 V vs. RHE, respectively, which indicated that more CO was desorbed at 1.22 V vs. RHE.

The current density vs. time curve was recorded as a function of oxidation period (Fig. 4b). The oxidation potential was fixed at 1.22 V vs. RHE while the oxidation period was varied from 2 to 20 s as shown in Fig. 4c. In the oxidation step, the current density initially decayed dramatically and then much more slowly after 20 s. This result indicates that capacitive and pseudo-capacitive reactions were almost terminated in 20 s. From the current density profiles, the recovery percentage was calculated by dividing the initial current density of the sixth cycle by that of the first cycle as shown in Fig. 4d. The average HCOO^−^ FE was also estimated via ^1^H NMR analysis of the catholyte after electrolysis.

The oxidation period critically affected the current density recovery and the HCOO^−^ selectivity. As the period increased from 2 to 10 s, the recovery percentage and the HCOO^−^ FE were enhanced from 23.0% to 95.2% and from 63.7% to 96.3%, respectively. These results show not only that CO is completely eliminated in 10 s, but also that full CO elimination leads to a high HCOO^−^ selectivity. When the period was prolonged to 20 s, the recovery percentage and the HCOO^−^ FE was almost unchanged. By additionally considering the electrical energy consumption, we found the optimal parameters of the oxidation step for fully and selectively removing CO with a small energy cost. Additionally, applying lower reduction potentials requires additional optimization of the oxidation step because CO poisoning proceeds at a higher rate. It can be observed that current density stepwise decreases to zero in the cyclic two-step electrolysis using −0.33 V vs. RHE (Supplementary Fig. 13). In this case, increasing the oxidation period or potential can be needed for stable formate production.

## Discussion

In summary, we have demonstrated that the inherent poisoning issue of Pd-based catalysts in electrochemical CO_2_ reduction reactions can be resolved via cyclic two-step electrolysis, an electrochemical method to apply the reduction and oxidation steps alternately. Though HCOO^−^ production and CO adsorption simultaneously occurred in the reduction step, the CO molecules were selectively oxidized in the anodic step. The reversibility difference between the concurrent reactions played a critical role in decoupling the reactions, and could be further secured by controlling the oxidation potential and period. From the cyclic two-step electrolysis, 97.8% selectivity and 100% current density stability for 45 h were accomplished toward HCOO^−^ production. In comparison, potentiostatic electrolysis showed that the current density and FE loss corresponded to 63.2 and 24.1%, respectively, in only 6 h. The performance degradation was caused by the increase in CO adsorption which not only blocks active sites, but also leads to dehydridation. We believe that our study can suggest an electrolysis method for solving poisoning issues in Pd-based electrochemical formate production.

## Methods

### Chemicals

Silver trifluoroacetate (98%) was purchased from Alfa Aesar. Commercial Pd/C (20 wt%) was purchased from Premetek. Ethylene glycol (99.5%) and methanol (99.8%) were purchased from Daejung Chemical & Metals. Carbon dioxide (99.999%) was purchased from the PSG Corporation. Sodium bicarbonate (99.5%) and sodium perchlorate monohydrate (98%) were purchased from Junsei Chemical.

### Catalyst synthesis and electrode preparation

The Pd_80_Ag_20_/C catalysts were synthesized via a microwave-assisted polyol method. Firstly, 11.3 mg of silver trifluoroacetate and 40 mg of 20 wt% Pd/C (Premetek) were dissolved in 50 ml of ethylene glycol. The input molar ratio of Ag/Pd was 0.67. Then, the precursor solution was sonicated for 30 m and heated by microwave irradiation at 200 W for 6 m. The resultant powder was centrifuged and washed with methanol. The powder was dried under vacuum at room temperature overnight. Pd/C catalysts as a control sample were synthesized with the same method without the silver precursor. For electrode preparation, 6 mg of catalyst powder was dispersed in 0.5 ml of ethanol with 40 μl of the neutralized Nafion solution, which was prepared by pH adjustment of 5 wt% Nafion dispersion solution (Sigma-Aldrich) with 0.1 M NaOH aqueous solution. A 40 μL aliquot of the catalyst dispersion was drop-coated on a glassy carbon electrode (Alfa Aesar). The active area was 0.5 cm^2^. Before CO_2_ reduction measurements of the Pd/C and Pd_80_Ag_20_/C catalysts, CV was scanned in the potential range from −0.35 to 1.10 V vs. Ag/AgCl at a rate of 50 mV s^−1^ in Ar-purged 0.1 M HClO_4_ solution for four cycles.

### Characterization

The TEM and STEM-EDS analyses were conducted using an FEI Talos F200X microscope. Powder XRD patterns were obtained with a D8 Advance X-ray diffractometer with Cu Kα radiation. ICP-OES measurement was performed on a Thermo iCAP 6000. For the sample preparation, 5 mg of Pd_80_Ag_20_/C powder was dissolved in 12 mL of 60% HNO_3_ and carbon black was separated using a filter paper (Whatman). The filtered solution was diluted with deionized water.

### Product analysis and stripping experiment

Electrolyses were conducted in a two-compartment gas-tight electrochemical cell with a piece of Nafion membrane (Nafion 117). The CV-treated catalyst electrodes and a piece of platinum foil were used as the working and counter electrodes, respectively. All potentials were controlled against an Ag/AgCl reference electrode (3.0 M NaCl, BASi) using a CHI 608 C potentiostat and converted to the RHE reference scale (*E*_RHE_ = *E*_Ag/AgCl_ + 0.210 V + 0.0591 V × *pH*). All potential values are given without *iR* correction. Before electrolysis, the catholyte, 0.5 M NaHCO_3_/0.5 M NaClO_4_, was purged with high-purity CO_2_ gas for at least 20 min. The electrolysis at various potentials was stopped when the passed charge was 0.8 C. After electrolysis, gas products in the headspace were extracted using a syringe (0.1 ml, Hamilton) and injected to a GC (Shimadzu) equipped with a packed Shincarbon ST 50/80 column. The gas mixtures were detected in a thermal conductivity detector (TCD) and a flame ionization detector (FID). For quantification, GC analyses were performed using standard gases with 10, 500 and 1000 ppm of H_2_ and CO (AirKorea Inc.). We then plotted the integrated area of H_2_ or CO signals with respect to the known concentrations for obtaining calibration curves (Supplementary Fig. 14). The H_2_ and CO concentrations in the sampled headspace gas were calculated based on the calibration curves. The concentration of produced HCOO^−^ was estimated using a 600 MHz NMR spectrometer (Avance 600, Bruker). For ^1^H NMR sample preparation, the catholyte (700 μL) containing the liquid product was mixed with the D_2_O solution (35 μL) of 10 mM dimethyl sulfoxide (DMSO) and 50 mM phenol as internal standards. For quantitative analysis, we prepared standard solutions by mixing the electrolyte (700 μL) containing 0, 10, 30, and 50 mM HCOO^−^ with the D_2_O solution (35 μL) containing 10 mM DMSO and 50 mM phenol. After ^1^H NMR measurements of the standards, the relative area of the HCOO^−^ signal respect to phenol was plotted as a function of HCOO^−^ concentration to obtain a calibration curve (Supplementary Fig. 14) as reported in a previous study^35^. The FE of the gas and liquid products was calculated by dividing the charge consumed to produce the specific amount of product by the total passed charge. The consumed charge was calculated by the equation: *C* × *V* × *F* × 2, where *C* is the concentration of H_2_ or CO or HCOO^−^, and *F* is the Faraday constant. *V* is the headspace volume for H_2_ and CO, while it is the catholyte volume for HCOO^−^. After electrolysis, anodic CV was scanned in the potential range from −0.65 to 0.65 V vs. Ag/AgCl at a scan rate of 10 mV s^−1^ in CO_2_-saturated 0.5 M NaHCO_3_/0.5 M NaClO_4_ for two cycles. To calculate the CO_strip_ and H_des_ charges, the area under the oxidation peaks was integrated and the charge caused by double layer charging and oxide formation was subtracted.

### Cyclic two-step electrolysis

The long-term cyclic two-step electrolysis displayed in Fig. 3 was conducted in a flow cell system as shown in Supplementary Fig. 12. The catholyte and anolyte, 0.5 M NaHCO_3_/0.5 M NaClO_4_, were circulated by a peristaltic pump between the electrolyte bottles and the electrochemical cell. The electrolyte in the bottle was purged with a CO_2_ gas during electrolysis. The CV-treated catalyst electrodes and a piece of platinum foil were used as the working and counter electrodes, respectively. The potential program per cycle is composed of the reduction step (−0.18 V vs. RHE for 9 min 50 s) and oxidation step ( + 1.22 V vs. RHE for 10 s). For analysis of gas products, CO_2_ gas was supplied to the cathode compartment and delivered to a GC (Younglin 6500 GC) at a flow rate of 20 mL min^−1^. The gas mixtures in a capillary column (Restek RT-Msieve 5 A) were transferred and detected in a pulsed discharge detector (PDD). The measurement of FEs for gas products was performed based on the following equation: FE_gas_ = (*I*_partial_/*I*_total_) × 100 = (*V* × *Q* × (*nFp*_*0*_/*RT*)/*I*_total_) × 100 where *I* is the current, *V* is the volume concentration of each gas product, *Q* is the gas flow rate, *n* is the number of electrons transferred to evolve a gas molecule, *F* is the Faraday constant, *p*_*0*_ is 1.01 bar, *R* is the ideal gas constant, and *T* is 298 K. The volume concentration was measured from the peak area of the GC spectrum, based on calibration with standard samples. The FE for HCOO^−^ production was measured by dividing the charge required to produce the specific amount of product by the cathodic charges passed during cyclic two-step electrolysis, based on ^1^H NMR analysis of the resultant catholyte after electrolysis. In the experiments of controlling the oxidation potential and period, CO_2_ gas was continuously supplied only to electrolyte bottles at a flow rate of 20 mL min^−1^.

## Supplementary information

Supplementary Information

Source data

## Data Availability

The authors declare that the main data supporting the findings of this study are available within the article and its Supplementary Information files. Extra data are available from the corresponding author upon request. The source data underlying Figs. 1d and 4d and Supplementary Figs. 1 and 11b are provided as a Source Data file.
