# Supplementary material for: Cyclic two-step electrolysis for stable electrochemical conversion of carbon dioxide to formate
Source: Nat Commun. 2019 Sep 2;10:3919. doi: 10.1038/s41467-019-11903-5 (PMC6718411; doi:10.1038/s41467-019-11903-5)
Supplement: Supplementary file 1 — Supplementary Information [file 41467_2019_11903_MOESM1_ESM.pdf]

Supplementary Information

Cyclic two-step electrolysis for stable electrochemical conversion of carbon dioxide to formate

Lee and Cho *et al.*

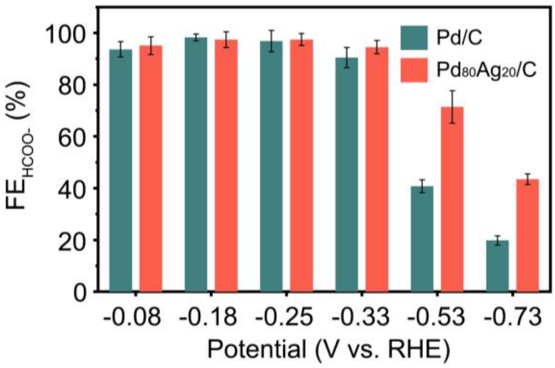

Supplementary Figure 1 The FEs for $HCOO^-$ production measured at various potentials on the Pd/C and $Pd_{80}Ag_{20}/C$ catalysts. Liquid product was analyzed using ^1H NMR after electrolysis at various potentials in a gas-tight cell. Error bars: mean±s.d. Source data are provided as a Source Data file.

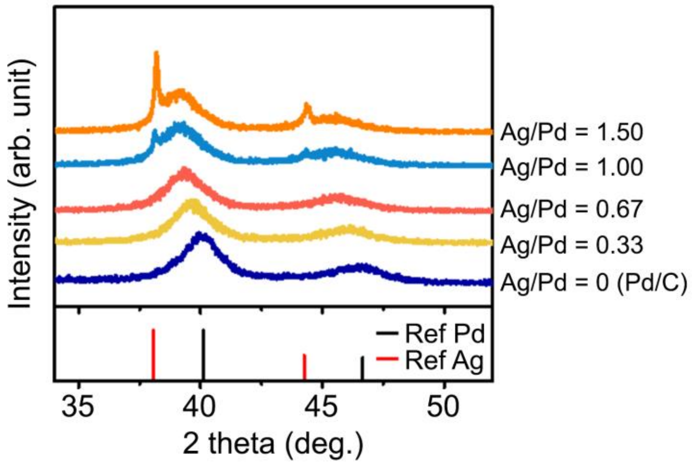

Supplementary Figure 2 XRD patterns of the powders synthesized with different molar ratios of Ag/Pd through the microwave-assisted polyol method. When the input ratio of Ag/Pd was 0.67, the XRD pattern showed the largest peak shift without the formation of pure Ag crystals.

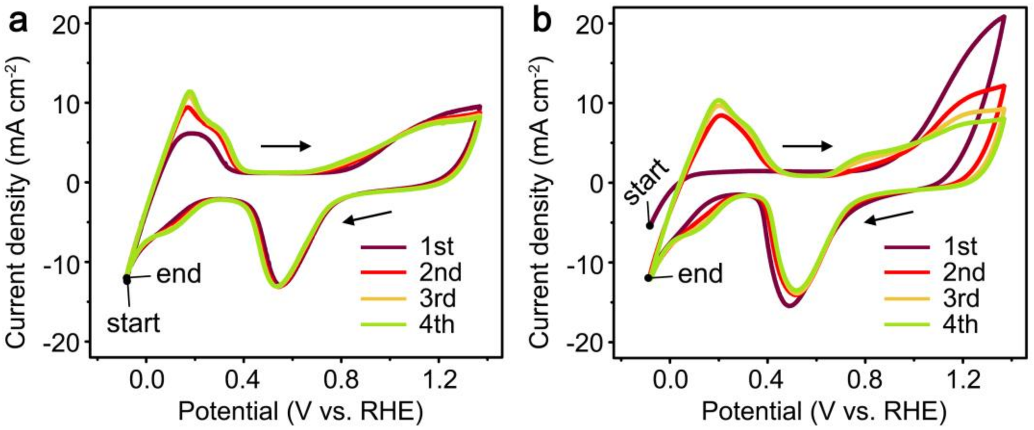

Supplementary Figure 3 CV pretreatment of as-synthesized NPs. The CV spectra of **a** Pd/C and **b** $Pd_{80}Ag_{20}$/C were scanned at a scan rate of 50 mV s^{-1} in Ar-purged 0.1 M $HClO_4$ solution for 4 cycles. For as-synthesized $Pd_{80}Ag_{20}$/C NPs, an irreversible oxidation peak at approximately 1.2 V vs. RHE was observed due to anodic dissolution of excessive Ag from the as-synthesized alloy.

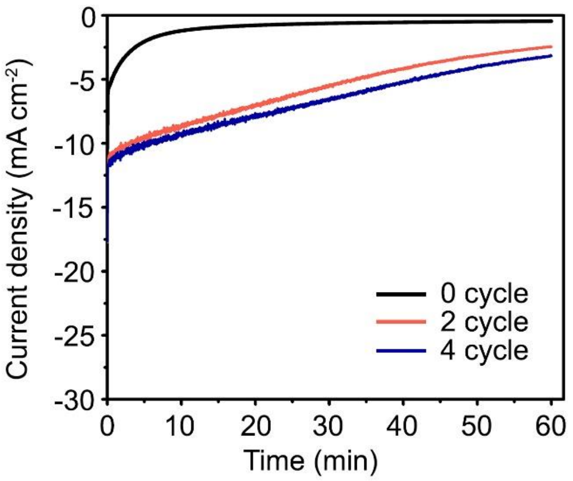

Supplementary Figure 4 Current density vs. time curve of $Pd_{80}Ag_{20}/C$ catalysts prepared with different cycle numbers of CV pretreatment. Chronoamperometry was conducted at -0.18 V vs. RHE in CO_2-saturated 0.5 M $NaHCO_3/NaClO_4$ solution.

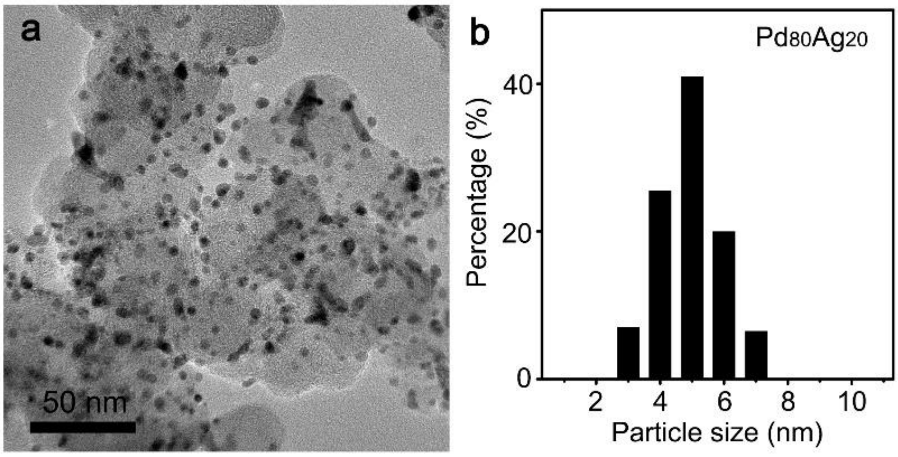

Supplementary Figure 5 TEM analysis results. **a** TEM image and **b** particle size distribution of $Pd_{80}Ag_{20}/C$ catalysts taken after CV pretreatment. The average particle size of the $Pd_{80}Ag_{20}/C$ catalysts was approximately 5.0 nm.

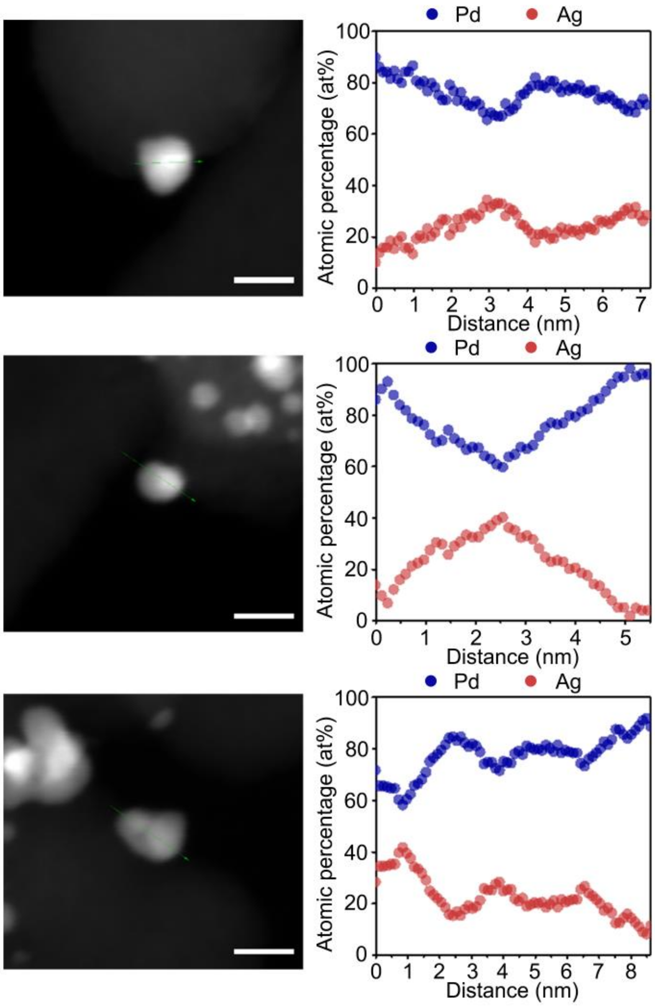

Supplementary Figure 6 HAADF-STEM images and STEM-EDS line scan profiles of the $Pd_{80}Ag_{20}/C$ catalysts taken after CV pretreatment. From EDS analysis, the average elemental ratio of Pd:Ag was measured to be 78.2:21.8. Scale bar = 8 nm.

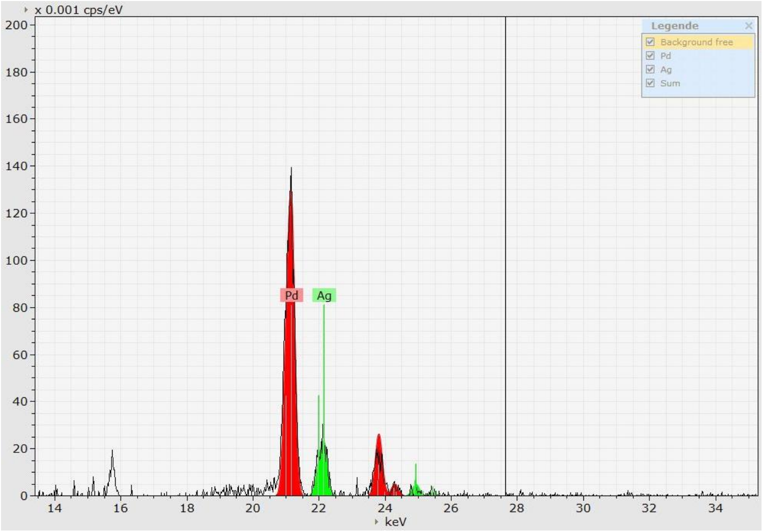

Supplementary Figure 7 Typical EDS spectrum of the $Pd_{80}Ag_{20}/C$ catalysts taken after CV pretreatment. The compositional information was obtained from the Kα and Kβ lines of Pd and Ag elements to sufficiently separate the EDS signals of Pd and Ag.

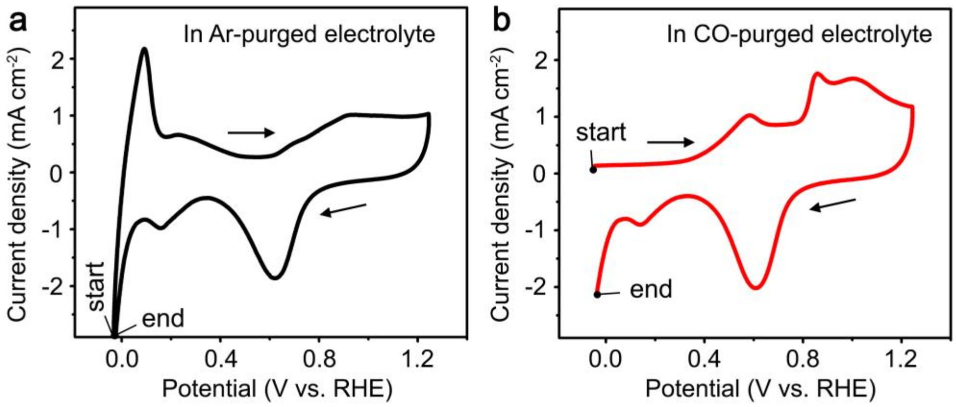

Supplementary Figure 8 Anodic CV spectra of Pd/C estimated after applying -0.20 V vs. RHE for 6 min in **a** Ar-purged and **b** CO-purged electrolyte. CV scans were conducted at a rate of 10 mV s^{-1}. By comparing the CV spectra, it can be identified that three additional peaks centered at approximately 0.60, 0.86 and 1.00 V vs. RHE appear in a CO-purged electrolyte condition.

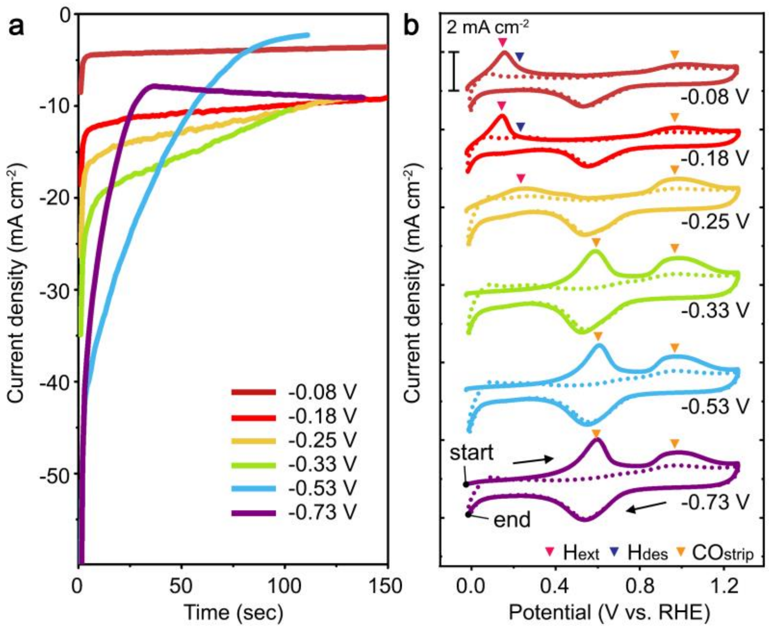

Supplementary Figure 9 Electrochemical surface analysis of Pd/C catalysts. **a** Current density vs. time curves at various potentials in a CO_2-saturated electrolyte. **b** CV curves recorded after electrolysis at various potentials. Solid and dotted lines are the first and second cycle of CV curves. In the first cycle, the anodic peaks related with H extraction from the lattice, H desorption from the surface, and CO stripping are indicated with pink, blue, and orange triangles, respectively.

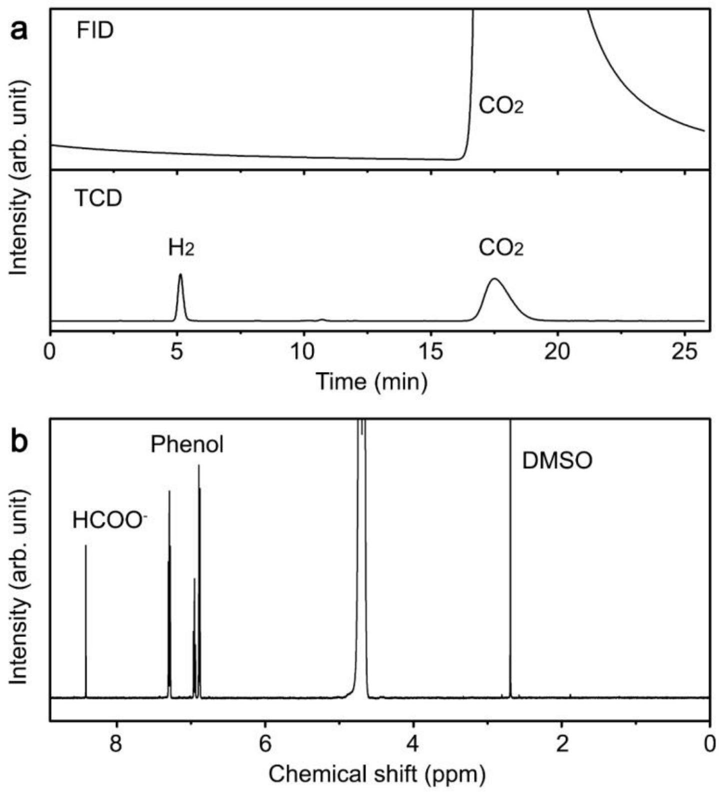

Supplementary Figure 10 Representative GC and ^{1}H NMR spectra of gas and liquid products. **a** The GC spectra were obtained by injecting a gas mixture in the headspace of a gas-tight cell after electrolysis at -0.53 V vs. RHE. The H_2 was detected in a TCD whereas CO was detected in an FID. **b** The ^{1}H NMR spectra were measured using the catholyte after electrolysis at -0.18 V vs. RHE. For ^{1}H NMR analysis, the catholyte was mixed with the D_2O solution containing DMSO and phenol as internal standards.

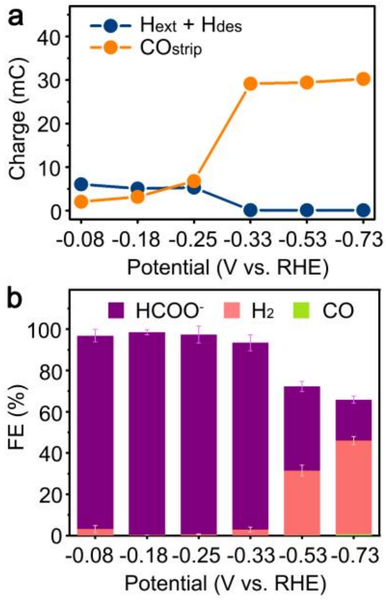

Supplementary Figure 11 Stripping charges and product selectivity of Pd/C. **a** CO_{strip} and H_{ext} + H_{des} charges measured from the CV curves of Pd/C shown in Supplementary Figure 9b. **b** The FEs for H_2 and $HCOO^-$ production measured from GC and ^{1}H NMR analysis after electrolysis on Pd/C at various potentials in a gas-tight cell. Error bars: mean±s.d. Source data are provided as a Source Data file.

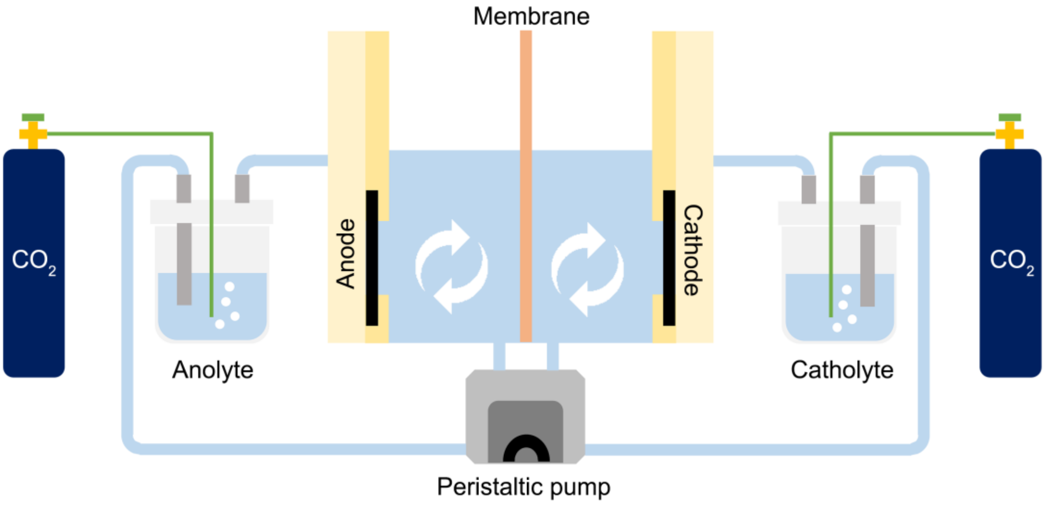

Supplementary Figure 12 Schematic diagram of a flow cell system composed of an electrochemical cell, a peristaltic pump and electrolyte bottles. The catholyte and anolyte were continuously circulated using a peristaltic pump between the electrolyte bottles and the electrochemical cell. The electrolyte was purged with a high-purity CO_2 gas during electrolysis.

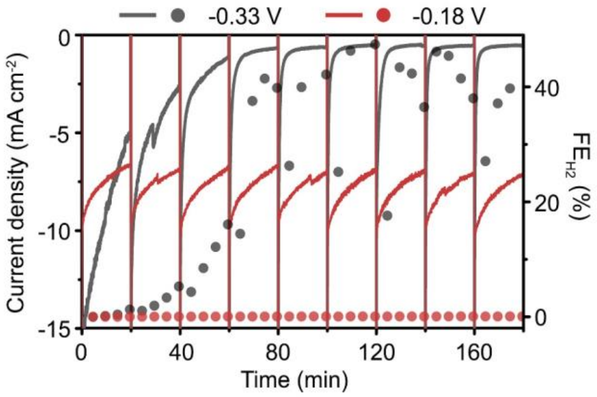

Supplementary Figure 13 Current density vs. time curves and H_2 production selectivity recorded during cyclic two-step electrolysis with different reduction potentials. In the reduction step, -0.18 or -0.33 V vs. RHE was applied with the reduction period fixed at 19 min 50 s. The oxidation potential and period were fixed at 1.22 V vs. RHE and 10 s, respectively. The experiments were conducted in a flow cell system where CO_2-saturated bicarbonate solution was continuously refreshed using a peristaltic pump.

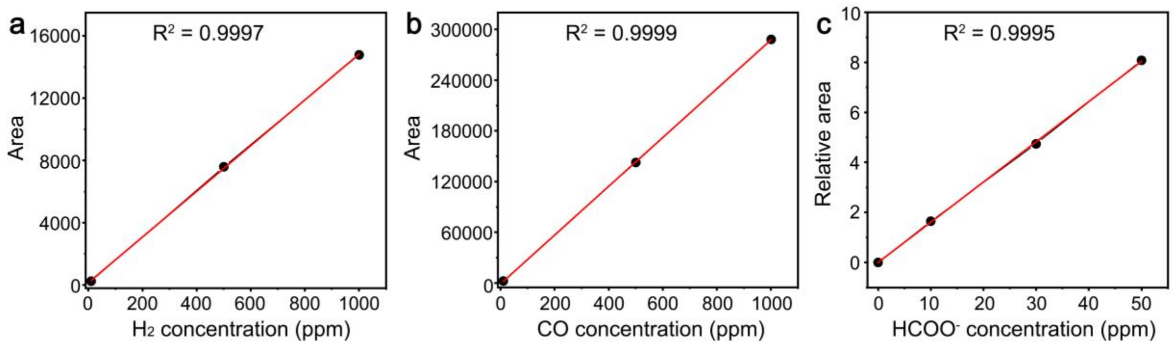

Supplementary Figure 14 Calibration curves for quantitative analysis. The calibration curves of **a** H_2 and **b** CO are obtained by performing GC analyses using standard gases containing 10, 500 and 1000 ppm of H_2 and CO. **c** The calibration curve of $HCOO^-$ is obtained by plotting the relative area of $HCOO^-$ signal respect to phenol as a function of $HCOO^-$ concentration, after ^{1}H MMR measurements of the standard samples containing 0, 10, 30 and 50 mM $HCOO^-$.

Supplementary Table 1 ICP-OES analysis result of $Pd_{80}Ag_{20}/C$ catalysts taken after CV pretreatment.

**Element**	**Atom%**
Pd	80.01
Ag	19.99

Supplementary Table 2 Summary on FEs for H_2 evolution, CO production, $HCOO^-$ production, H adsorption/absorption and CO adsorption on $Pd_{80}Ag_{20}/C$ catalysts. The FEs for H_2, CO and $HCOO^-$ production were measured from GC and ^1H NMR analysis after electrolysis at various potentials. The FEs for CO adsorption and H adsorption/absorption were calculated by dividing the CO_{strip} and H_{ext} + H_{des} charges by passed charge, respectively.

Potential (V vs. RHE)	FE for H_2 production (%)	FE for CO production (%)	FE for $HCOO^-$ production (%)	FE for H adsorption/absorption (%)	FE for CO adsorption (%)	Total FE (%)
-0.08	1.4	0.0	95.1	0.5	0.3	97.3
-0.18	0.1	0.0	97.4	0.5	0.4	98.4
-0.25	0.2	0.0	97.4	1.0	0.4	99.0
-0.33	0.7	0.2	94.5	1.3	0.5	97.2
-0.53	21.3	0.2	71.4	1.1	2.4	96.4
-0.73	42.7	0.1	43.5	0.3	5.5	92.1

Supplementary Table 3 Reported electrochemical $HCOO^-$ production performance on Pd-based catalysts. The reported data were extracted or collected from the literature that are listed in the Supplementary References.

Electrolysis method	Catalyst	Electrolyte	Potential (V vs. RHE)	Electrolysis duration	FE_{HCOO-} (%)	Initial current density (mA cm^{-2})	Final current density (mA cm^{-2})	Ref
Potentiostatic electrolysis in an H-cell	Pd/C	0.5 M $NaHCO_3$	-0.25	1 h	98.0	4.2	3.0	1
Potentiostatic electrolysis in an H-cell	High-index Pd	0.5 M $KHCO_3$	-0.20	50 min	97.0	20.0	17.0	2
Potentiostatic electrolysis in an H-cell	B-doped Pd/C	0.1 M $KHCO_3$	-0.50	5 h	55.0	6.5	3.6	3
Potentiostatic electrolysis in an H-cell	6.5 nm Pd NPs	0.5 M $NaHCO_3$	-0.20	1 h	94.0	6.0	2.0	4
Potentiostatic electrolysis in an H-cell	$Pd_{70}Pt_{30}/C$	pH 6.8 phosphate buffer	-0.40	2 h	57.6	7.0	3.5	5
Potentiostatic electrolysis in an H-cell	3.7 nm Pd NPs	1.0 M $KHCO_3$	-0.20	1 h	98.0	18.2	13.9	6
Potentiostatic electrolysis in an H-cell	Electrodeposited Pd	0.1 M $KHCO_3$	-0.40	20 min	55.8	2.0	1.4	7
Potentiostatic electrolysis in an H-cell	Pd foil modified by NHC ligand	0.5 M $KHCO_3$	-0.57	6 h	<80.0	2.2	2.0	8
Potentiostatic electrolysis in an H-cell	Pd/Pt/C	1.0 M $KHCO_3$	-0.20	1 h	91.9	29.7	19.4	9
Cyclic two-step electrolysis in a flow cell	$Pd_{80}Ag_{20}/C$	0.5 M $NaHCO_3$ /0.5 M $NaClO_4$	-0.18 V for 590 s / 1.22 V for 10 s	45 h	97.8	11.0	11.0	Our work

Supplementary References

1. Min, X. & Kanan, M. W. Pd-Catalyzed electrohydrogenation of carbon dioxide to formate: high mass activity at low overpotential and identification of the deactivation pathway. *J. Am. Chem. Soc.* **137,** 4701–4708 (2015).
2. Klinkova, A. *et al.* Rational design of efficient palladium catalysts for electroreduction of carbon dioxide to formate. *ACS Catal.* **6,** 8115–8120 (2016).
3. Jiang, B., Zhang, X.-G., Jiang, K., Wu, D.-Y. & Cai, W.-B. Boosting formate production in electrocatalytic CO_2 reduction over wide potential window on Pd Surfaces. *J. Am. Chem. Soc.* **140,** 2880–2889 (2018).
4. Rahaman, M., Dutta, A. & Broekmann, P. Size-dependent activity of palladium nanoparticles: efficient conversion of CO_2 into formate at low overpotentials. *ChemSusChem* **10,** 1733–1741 (2017).
5. Kortlever, R., Peters, I., Koper, S. & Koper, M. T. M. Electrochemical CO_2 reduction to formic acid at low overpotential and with high Faradaic efficiency on carbon-supported bimetallic Pd-Pt nanoparticles. *ACS Catal.* **5,** 3916–3923 (2015).
6. Gao, D. *et al.* Switchable CO_2 electroreduction via engineering active phases of Pd nanoparticles. *Nano Res.* **10,** 2181–2191 (2017).
7. Zhou, F., Li, H., Fournier, M. & MacFarlane, D. R. Electrocatalytic CO_2 reduction to formate at low overpotentials on electrodeposited Pd Films: stabilized performance by suppression of CO formation. *ChemSusChem* **10,** 1509–1516 (2017).
8. Cao, Z. *et al.* Chelating N-heterocyclic carbene ligands enable tuning of electrocatalytic CO_2 reduction to formate and carbon monoxide: surface organometallic chemistry. *Angew. Chem. Int. Ed.* **57,** 4981–4985 (2018).
9. Cai, F. *et al.* Effect of metal deposition sequence in carbon-supported Pd–Pt catalysts on activity towards CO_2 electroreduction to formate. *Electrochem. Commun.* **76,** 1–5 (2017).
